# VarSight: prioritizing clinically reported variants with binary classification algorithms

**DOI:** 10.1186/s12859-019-3026-8

**Published:** 2019-10-15

**Authors:** James M. Holt, Brandon Wilk, Camille L. Birch, Donna M. Brown, Manavalan Gajapathy, Alexander C. Moss, Nadiya Sosonkina, Melissa A. Wilk, Julie A. Anderson, Jeremy M. Harris, Jacob M. Kelly, Fariba Shaterferdosian, Angelina E. Uno-Antonison, Arthur Weborg, Undiagnosed Diseases Network, Maria T. Acosta, Margaret Adam, David R. Adams, Pankaj B. Agrawal, Mercedes E. Alejandro, Patrick Allard, Justin Alvey, Laura Amendola, Ashley Andrews, Euan A. Ashley, Mahshid S. Azamian, Carlos A. Bacino, Guney Bademci, Eva Baker, Ashok Balasubramanyam, Dustin Baldridge, Jim Bale, Michael Bamshad, Deborah Barbouth, Gabriel F. Batzli, Pinar Bayrak-Toydemir, Anita Beck, Alan H. Beggs, Gill Bejerano, Hugo J. Bellen, Jimmy Bennet, Beverly Berg-Rood, Raphael Bernier, Jonathan A. Bernstein, Gerard T. Berry, Anna Bican, Stephanie Bivona, Elizabeth Blue, John Bohnsack, Carsten Bonnenmann, Devon Bonner, Lorenzo Botto, Lauren C. Briere, Elly Brokamp, Elizabeth A. Burke, Lindsay C. Burrage, Manish J. Butte, Peter Byers, John Carey, Olveen Carrasquillo, Ta Chen Peter Chang, Sirisak Chanprasert, Hsiao-Tuan Chao, Gary D. Clark, Terra R. Coakley, Laurel A. Cobban, Joy D. Cogan, F. Sessions Cole, Heather A. Colley, Cynthia M. Cooper, Heidi Cope, William J. Craigen, Michael Cunningham, Precilla D’Souza, Hongzheng Dai, Surendra Dasari, Mariska Davids, Jyoti G. Dayal, Esteban C. Dell’Angelica, Shweta U. Dhar, Katrina Dipple, Daniel Doherty, Naghmeh Dorrani, Emilie D. Douine, David D. Draper, Laura Duncan, Dawn Earl, David J. Eckstein, Lisa T. Emrick, Christine M. Eng, Cecilia Esteves, Tyra Estwick, Liliana Fernandez, Carlos Ferreira, Elizabeth L. Fieg, Paul G. Fisher, Brent L. Fogel, Irman Forghani, Laure Fresard, William A. Gahl, Ian Glass, Rena A. Godfrey, Katie Golden-Grant, Alica M. Goldman, David B. Goldstein, Alana Grajewski, Catherine A. Groden, Andrea L. Gropman, Sihoun Hahn, Rizwan Hamid, Neil A. Hanchard, Nichole Hayes, Frances High, Anne Hing, Fuki M. Hisama, Ingrid A. Holm, Jason Hom, Martha Horike-Pyne, Alden Huang, Yong Huang, Rosario Isasi, Fariha Jamal, Gail P. Jarvik, Jeffrey Jarvik, Suman Jayadev, Yong-hui Jiang, Jean M. Johnston, Lefkothea Karaviti, Emily G. Kelley, Dana Kiley, Isaac S. Kohane, Jennefer N. Kohler, Deborah Krakow, Donna M. Krasnewich, Susan Korrick, Mary Koziura, Joel B. Krier, Seema R. Lalani, Byron Lam, Christina Lam, Brendan C. Lanpher, Ian R. Lanza, C. Christopher Lau, Kimberly LeBlanc, Brendan H. Lee, Hane Lee, Roy Levitt, Richard A. Lewis, Sharyn A. Lincoln, Pengfei Liu, Xue Zhong Liu, Nicola Longo, Sandra K. Loo, Joseph Loscalzo, Richard L. Maas, Ellen F. Macnamara, Calum A. MacRae, Valerie V. Maduro, Marta M. Majcherska, May Christine V. Malicdan, Laura A. Mamounas, Teri A. Manolio, Rong Mao, Kenneth Maravilla, Thomas C. Markello, Ronit Marom, Gabor Marth, Beth A. Martin, Martin G. Martin, Julian A. Martínez-Agosto, Shruti Marwaha, Jacob McCauley, Allyn McConkie-Rosell, Colleen E. McCormack, Alexa T. McCray, Heather Mefford, J. Lawrence Merritt, Matthew Might, Ghayda Mirzaa, Eva Morava-Kozicz, Paolo M. Moretti, Marie Morimoto, John J. Mulvihill, David R. Murdock, Avi Nath, Stan F. Nelson, John H. Newman, Sarah K. Nicholas, Deborah Nickerson, Donna Novacic, Devin Oglesbee, James P. Orengo, Laura Pace, Stephen Pak, J. Carl Pallais, Christina GS. Palmer, Jeanette C. Papp, Neil H. Parker, John A. Phillips III, Jennifer E. Posey, John H. Postlethwait, Lorraine Potocki, Barbara N. Pusey, Aaron Quinlan, Wendy Raskind, Archana N. Raja, Genecee Renteria, Chloe M. Reuter, Lynette Rives, Amy K. Robertson, Lance H. Rodan, Jill A. Rosenfeld, Robb K. Rowley, Maura Ruzhnikov, Ralph Sacco, Jacinda B. Sampson, Susan L. Samson, Mario Saporta, C. Ron Scott, Judy Schaechter, Timothy Schedl, Kelly Schoch, Daryl A. Scott, Lisa Shakachite, Prashant Sharma, Vandana Shashi, Jimann Shin, Rebecca Signer, Catherine H. Sillari, Edwin K. Silverman, Janet S. Sinsheimer, Kathy Sisco, Kevin S. Smith, Lilianna Solnica-Krezel, Rebecca C. Spillmann, Joan M. Stoler, Nicholas Stong, Jennifer A. Sullivan, Angela Sun, Shirley Sutton, David A. Sweetser, Virginia Sybert, Holly K. Tabor, Cecelia P. Tamburro, Queenie K.-G. Tan, Mustafa Tekin, Fred Telischi, Willa Thorson, Cynthia J. Tifft, Camilo Toro, Alyssa A. Tran, Tiina K. Urv, Matt Velinder, Dave Viskochil, Tiphanie P. Vogel, Colleen E. Wahl, Stephanie Wallace, Nicole M. Walley, Chris A. Walsh, Melissa Walker, Jennifer Wambach, Jijun Wan, Lee-kai Wang, Michael F. Wangler, Patricia A. Ward, Daniel Wegner, Mark Wener, Monte Westerfield, Matthew T. Wheeler, Anastasia L. Wise, Lynne A. Wolfe, Jeremy D. Woods, Shinya Yamamoto, John Yang, Amanda J. Yoon, Guoyun Yu, Diane B. Zastrow, Chunli Zhao, Stephan Zuchner, Elizabeth A. Worthey

**Affiliations:** 1 HudsonAlpha Institute for Biotechnology, Software Development and Informatics, 601 Genome Way, Huntsville, 35806 USA; 2 University of Alabama at Birmingham, Department of Genetics, 720 20th Street South, Birmingham, 35294 USA

**Keywords:** Clinical genome sequencing, Variant prioritization, Binary classification

## Abstract

**Background:**

When applying genomic medicine to a rare disease patient, the primary goal is to identify one or more genomic variants that may explain the patient’s phenotypes. Typically, this is done through annotation, filtering, and then prioritization of variants for manual curation. However, prioritization of variants in rare disease patients remains a challenging task due to the high degree of variability in phenotype presentation and molecular source of disease. Thus, methods that can identify and/or prioritize variants to be clinically reported in the presence of such variability are of critical importance.

**Methods:**

We tested the application of classification algorithms that ingest variant annotations along with phenotype information for predicting whether a variant will ultimately be clinically reported and returned to a patient. To test the classifiers, we performed a retrospective study on variants that were clinically reported to 237 patients in the Undiagnosed Diseases Network.

**Results:**

We treated the classifiers as variant prioritization systems and compared them to four variant prioritization algorithms and two single-measure controls. We showed that the trained classifiers outperformed all other tested methods with the best classifiers ranking 72% of all reported variants and 94% of reported pathogenic variants in the top 20.

**Conclusions:**

We demonstrated how freely available binary classification algorithms can be used to prioritize variants even in the presence of real-world variability. Furthermore, these classifiers outperformed all other tested methods, suggesting that they may be well suited for working with real rare disease patient datasets.

**Electronic supplementary material:**

The online version of this article (10.1186/s12859-019-3026-8) contains supplementary material, which is available to authorized users.

## Background

Genome and exome sequencing are both currently being used as molecular diagnostic tools for patients with rare, undiagnosed diseases [1–3]. Typically, these technologies are applied clinically by following workflows consisting of blood draw, sequencing, alignment, variant calling, variant annotation, variant filtering, and variant prioritization [4, 5]. Then, clinical analysts usually perform the more manual processes of inspecting and then reporting variants based on a set of patient phenotypes from the referring doctor.

In general, commonly used pipelines exist for the steps from sequencing through variant calling [6, 7]. Despite differences in performance, most of these pipelines are relatively uniform in that they start with the same inputs (i.e. read files, commonly FASTQ format) and produce the same outputs (i.e. a set of variants, commonly Variant Call Format). In contrast, methods for variant annotation and/or variant filtering are quite diverse [8–11]. These methods use a wide range of annotation sources including but not limited to population allele frequencies [12], conservation scores [13–15], haploinsufficiency scores [16, 17], deleteriousness scores [17, 18], transcript impact scores [19–23], and previously associated disease annotation [24–26]. Variant prioritization is also quite diverse with some methods relying only on the variant annotations to prioritize variants [9] and some relying only on patient phenotype to rank the variants [27–30]. There are also methods which combine both variant annotations and phenotype score to rank the variants [31–34], a selection of which are benchmarked on the same simulated datasets in [35].

Given a prioritized list of variants, analysts manually inspect each one and curate a subset to ultimately report to the ordering physician. Unfortunately, manual curation is a time consuming process where analysts must inspect each variant while maintaining a mental picture of the patient’s phenotype. One group reported an average of 600 variants per case analyzed by two people (one analyst and one director) over three hours, meaning a throughput of ≈100 variants per man-hour [36]. If causative variants can be identified earlier due to a high rank from prioritization, it’s possible that the full filtered variant list can be short-circuited, reducing the total number of variants reviewed and therefore the time to analyze a case. Additionally, accurate prioritization is a step towards the ultimate goal of fully automating the analysis of the sequencing data for rare disease patients.

One of the issues with previously published ranking methods is that they were primarily tested on simulated datasets with known, single-gene, pathogenic variants injected into real or simulated background genomic datasets. Additionally, when phenotype terms were used, they tended to select all matching phenotype terms for the simulated disease and then inject/remove a few terms (typically 2-3) in order to provide some variability. In practice, rare disease patients often have much more variability in their phenotype terms for a wide variety of reasons such as multiple genetic diseases, variability in disease presentation, phenotypes of non-genetic origin, and/or variability in the standards describing a phenotype.

In this paper, we focus on real patient data from the multi-site collaboration of the Undiagnosed Diseases Network (UDN) [1]. Patients accepted into the UDN are believed to have rare, undiagnosed diseases of genetic origin. Because the UDN is not focused on a single particular disease, the patient population has a diverse range of phenotypes represented. Additionally, the exact phenotype terms associated to an individual patient are highly variable for the reasons described above. Because the UDN is a research collaboration, there is also variability in reported variants that range in pathogenicity from “variant of uncertain significance” (VUS) through “pathogenic” as defined by the ACMG guidelines [37]. The summation of this real-world variation means that accurately identifying and/or prioritizing variants is challenging due to uncertainty and variation in phenotype inputs and variation in pathogenicity of reported variant outputs.

## Methods

### Overview

We tested the application of classification algorithms for identifying clinically reported variants in real world patients in two ways: 1) predicting whether a variant observed by an analyst would be clinically reported and 2) prioritizing all variants seen by the clinical analysts. In particular, we focused our analyses on real patients with a diverse collection of rare, undiagnosed diseases that were admitted to the Undiagnosed Diseases Network (UDN) [1]. We limited our patients to those who received whole genome sequencing and received at least one primary variant (i.e. not secondary or incidental) on their clinical report. We extracted data directly from the same annotation and filtering tool used by the analysts in order to replicate their data view of each variant in a patient. Additionally, we incorporated phenotype information into the models using two scoring systems that are based on ranking genes by their association to a set of patient phenotypes. Finally, each variant was either labeled as “returned” or “not returned” depending on whether it was ultimately reported back to the clinical site.

Given the above variant information, we split the data into training and testing sets for measuring the performance of classifiers to predict whether a variant would be clinically reported or not. We tested four classifiers that are readily available in the *sklearn* [38] and *imblearn* [39] Python modules. Of note, our focus was not on picking the “best” classifier, but rather on analyzing their overall ability to handle the variability of real-world patient cases from the UDN.

Each classifier calculated probabilities of a variant belonging to the “returned” class, allowing us to measure their performance as both a classifier and a prioritization/ranking system. After tuning each classifier, we generated summaries of the performance of each method from both a binary classification perspective and a variant prioritization perspective. Additionally, we tested four publicly available variant prioritization algorithms and two single-value ranking methods for comparison. All of the scripts to train classifiers, test classifiers, and format results are contained in the VarSight repository. A visualization of the workflow for gathering features, training the models, and testing the models can be found in the Additional file 1.

### Data sources

All samples were selected from the cohort of Undiagnosed Diseases Network (UDN) [1] genome sequencing samples that were sequenced at HudsonAlpha Institute for Biotechnology (HAIB). In short, the UDN accepts patients with rare, undiagnosed diseases that are believed to have a genetic origin. The UDN is not restricted to a particular disease, so there are a diverse set of diseases and phenotypes represented across the whole population. The phenotypes annotated to a patient are also variable compared to simulated datasets for a variety of reasons including: 1) patients may have multiple genetic diseases, 2) phenotype collection is done at seven different clinical sites leading to differences in the standards of collection, 3) patients may exhibit more or fewer phenotypes than are associated with the classic disease presentation, and 4) patients may have phenotypes of non-genetic origin such as age- or pathogen-related phenotypes. For more details on the UDN, we refer the reader to Ramoni et al., 2017 [1].

DNA for these UDN patients was prepared from whole blood samples (with few exceptions) and sequenced via standard operation protocols for use as a Laboratory-Developed Test in the HAIB CAP/CLIA lab. The analyses presented in this paper are based on data that is or will be deposited in the dbGaP database under dbGaP accession phs001232.v1.p1 by the UDN.

### Alignment and variant calling

After sequencing, we followed GATK best practices [40] to align to the GRCh37 human reference genome with BWA-mem [41]. Aligned sequences were processed via GATK for base quality score recalibration, indel realignment, and duplicate removal. Finally, SNV and indel variants were joint genotyped, again following GATK best practices [40]. The end result of this pipeline is one Variant Call Format (VCF) file per patient sample. This collection of VCF files is used in the following sections.

### Variant annotation and filtering

After VCF generation, the clinical analysts followed various published recommendations (e.g. [4, 5]) to annotate and filter variants from proband samples. For variant annotation and filtering, we used the same tool that our analysts used during their initial analyses. The tool, Codicem [42], loads patient variants from a VCF and annotates the variants with over fifty annotations that the analysts can use to interpret pathogenicity. These annotations include: variant level annotations such as CADD [18], conservation scores [13, 14], and population frequencies [12]; gene level annotations such as haploinsufficiency scores [16, 17], intolerance scores [15], and disease associations [24–26]; and transcript level annotations such as protein change scores [19–22] and splice site impact scores [23]. Additionally, if the variant has been previously curated in another patient through Human Gene Mutation Database (HGMD) or ClinVar [24, 26], those annotations are also made available to the analysts.

Codicem also performs filtering for the analysts to reduce the number of variants that are viewed through a standard clinical analysis. We used the latest version of the primary clinical filter for rare disease variants to replicate the standard filtering process for patients in the UDN. In short, the following criteria must be met for a variant to pass through the clinical filter: sufficient total read depth, sufficient alternate read depth, low population frequency, at least one predicted effect on a transcript, at least one gene-disease association, and to not be a known, common false-positive from sequencing. In general, the filter reduces the number of variants from the order of millions to hundreds (anecdotally, roughly 200-400 variants per proband after filtering). For details on the specific filter used, please refer to Additional file 1.

### Phenotype annotation

The Codicem annotations are all agnostic of the patient phenotype. As noted earlier, we do not expect the patient phenotypes to exactly match the classic disease presentation due to the variety and complexity of diseases, phenotypes, and genetic heritage tied to UDN patients. Despite this, we made no effort to alter or condense the set of phenotypes provided by the corresponding clinical sites. In order to incorporate patient phenotype information, we used two distinct methods to rank genes based on the Human Phenotype Ontology (HPO) [43]. We then annotated each variant with the best scores from their corresponding gene(s).

The first method uses phenotype-to-gene annotations provided by the HPO to calculate a cosine score [44] between the patient’s phenotypes and each gene. Given *P* terms in the HPO, this method builds a binary, *P*-dimensional vector for each patient such that only the phenotype terms (including ancestral terms in the ontology) associated with the patient are set to 1, and all other terms are set to 0. Similarly, a *P*-dimensional vector for each gene is built using the phenotype-to-gene annotations. Then, the cosine of the angle between the patient vector and each gene vector is calculated as a representation of similarity. This method tends to be more conservative because it relies solely on curated annotations from the HPO.

The second method, an internally-developed tool called PyxisMap [30], uses the same phenotype-to-gene annotations from the HPO, but adds in automatically text-mined annotations from NCBI’s PubTator [45] and performs a Random-Walk with Restart [46] on the ontology graph structure. The PyxisMap method has the added benefit of incorporating gene-phenotype connections from recent papers that have not been manually curated into the HPO, but it also tends to make more spurious connections due to the imprecision of the text-mining from PubTator. Each method generates a single numerical feature that is used in the following analyses.

### Patient selection

In the clinical analysis, each patient was fully analyzed by one director and one analyst. After the initial analysis, the full team of directors and analysts review flagged variants and determine their reported pathogenicity. In our analysis, we focused on variants that were clinically reported as “primary”, meaning the team of analysts believed the variant to be directly related to the patient’s phenotype. Note that secondary and/or incidental findings are specifically not included in this list. The team of analysts assigned each primary variant a classification of variant of uncertain significance (VUS), likely pathogenic, or pathogenic adhering to the recommendations in the American College of Medical genetics (ACMG) guidelines for variant classification [37].

We required the following for each proband sample included in our analyses: 1) at least one clinically reported primary variant that came through the primary clinical filter (i.e. it was not found through some other targeted search) and 2) a set of phenotypes annotated with Human Phenotype Ontology [43] terms using the Phenotips software [47]. At the time of writing, this amounted to 378 primary-reported variants and 87819 unreported variants spanning a total of 237 proband samples.

### Feature selection

For the purposes of classification, all annotations needed to be cleaned, reformatted, and stored as numerical features. For single-value numerical annotations (e.g. float values like CADD), we simply copied the annotation over as a single value feature. Missing annotations were assigned a default value that was outside the expected value range for that feature. Additionally, these default values were always on the less impactful side of the spectrum (e.g. a default conservation score would err on the side of not being conserved). The one exception to this rule was for variant allele frequencies where a variant absent from a database was considered to have an allele frequency of 0.0. For multi-value numerical annotations, we reduced the values (using minimum or maximum) to a single value corresponding to the “worst” value (i.e. most deleterious value, most conserved value, etc.) that was used as the feature.

For categorical data, we relied on bin-count encoding to store the features. We chose to bin-count because there are many annotations where multiple categorical labels may be present at different quantities. For example, a single ClinVar variant may have multiple entries where different sites have selected different levels of pathogenicity. In this situation, we desired to capture not only the categorical label as a feature, but also the number of times that label occurred in the annotations.

After converting all annotations to numerical features, we had a total of 95 features per variant. We then pruned down to only the top 20 features using univariate feature selection (specifically the SelectKBest method of *sklearn* [38]). This method evaluates how well an individual feature performs as a classifier and keeps only the top 20 features for the full classifiers. Note that only the training set was used to select the top features and that selection was later applied to the testing set prior to final evaluation. Table 1 shows the list of retained features ordered by feature importance after training. Feature importance was derived from the random forest classifiers which automatically report how important each feature was for classification. The entire set of annotations along with descriptions of how each was processed prior to feature selection are detailed in the Additional file 1.

**Table 1:** Feature selection

Feature label	RF(sklearn)	BRF(imblearn)
HPO-cosine	0.2895	0.2471
PyxisMap	0.2207	0.2079
CADD Scaled	0.1031	0.1007
phylop100 conservation	0.0712	0.0817
phylop conservation	0.0641	0.0810
phastcon100 conservation	0.0572	0.0628
GERP rsScore	0.0357	0.0416
HGMD assessment type_DM	0.0373	0.0344
HGMD association confidence_High	0.0309	0.0311
Gnomad Genome total allele count	0.0192	0.0322
ClinVar Classification_Pathogenic	0.0228	0.0200
ADA Boost Splice Prediction	0.0081	0.0109
Random Forest Splice Prediction	0.0077	0.0105
Meta Svm Prediction_D	0.0088	0.0092
PolyPhen HV Prediction_D	0.0075	0.0071
Effects_Premature stop	0.0049	0.0057
SIFT Prediction_D	0.0026	0.0056
PolyPhen HD Prediction_D	0.0025	0.0049
Effects_Possible splicing modifier	0.0029	0.0035
ClinVar Classification_Likely Pathogenic	0.0034	0.0020

This table shows the top 20 features that were used to train the classifiers ordered from most important to least important. After training, the two random forest classifiers report the importance of each feature in the classifier (total is 1.00 per classifier). We average the two importance values, and order them from most to least important. Feature labels with an ‘_’ represent a single category of a multi-category feature (i.e. “HGMD assessment type_DM” means the “DM” bin-count feature from the “HGMD assessment type” annotation in Codicem)

### Classifier training and tuning

As noted earlier, there are generally hundreds of variants per proband that pass the filter, but only a few are ever clinically reported. Across all 237 proband samples, there were a total of 378 clinically reported variants and another 87819 variants that were seen but not reported. As a result, there is a major imbalance in the number of true positives (variants clinically reported) and true negatives (variants seen, but not clinically reported).

We split the data into training and test sets on a per-proband basis with the primary goal of roughly balancing the total number of true positives in each set. Additionally, the cases were assigned to a particular set by chronological order of analysis in order to reduce any chronological biases that may be introduced by expanding scientific knowledge (i.e. there are roughly equal proportions of “early” or “late” proband samples from the UDN in each set). In the training set, there were a total of 189 returned variants and 44593 not returned variants spanning 120 different probands. In the test set, there were a total of 189 returned variants and 43226 not returned variants spanning 117 different probands. In our results, the returned test variants are further stratified by their reported levels of pathogenicity.

We then selected four publicly available binary-classification models that are capable of training on imbalanced datasets: the RandomForest model by *sklearn* [38], the LogisticRegression model by *sklearn*, the BalancedRandomForest model by *imblearn* [39], and the EasyEnsembleClassifier model by *imblearn*. These classifiers were chosen for three main reasons: 1) their ability to handle imbalanced data (i.e. far more unreported variants than reported variants), 2) their ability to scale to the size of the training and testing datasets, and 3) they are freely available implementations that can be tuned, trained, and tested with relative ease in the same Python framework. The two random forest classifiers build collections of decision trees that weight each training input by its class frequency. Logistic regression calculates the probability of a value belonging to a particular class, again weighting by the class frequency. In contrast to the other three tested methods, the ensemble classification balances the training input using random under-sampling and then trains an ensemble of AdaBoost learners. For more details on each classifier, please refer to the *sklearn* and *imblearn* documentations [38, 39].

Initially, we also tested the support vector classifier by *sklearn* (SVC), the multi-layer perceptron by *sklearn* (MLPClassifier), and the random under-sampling AdaBoost classifier by *imblearn* (RUSBoostClassifier). Each of these was excluded from our results due to, respectively, scaling issues with the training size, failure to handle the data imbalance, and overfitting to the training set. While we did not achieve positive results using these three implementations, it may be possible to use the methods through another implementation.

For each of our tested classifiers, we selected a list of hyperparameters to test and tested each possible combination of those hyperparameters. For each classifier and set of hyperparameters, we performed stratified 10-fold cross validation on the training variants and recorded the balanced accuracy (i.e. weighted accuracy based on inverse class frequency) and the F1 scores (i.e. harmonic mean between precision and recall). For each classifier type, we saved the hyperparameters and classifier with the best average F1 score (this is recommended for imbalanced datasets). These four tuned classifiers were then trained on the full training set and tested against the unseen set of test proband cases. The set of hyperparameters tested along with the highest performance setting for each hyperparameter can be found in the Additional file 1.

## Results

### Classifier statistics

**Fig. 1:**
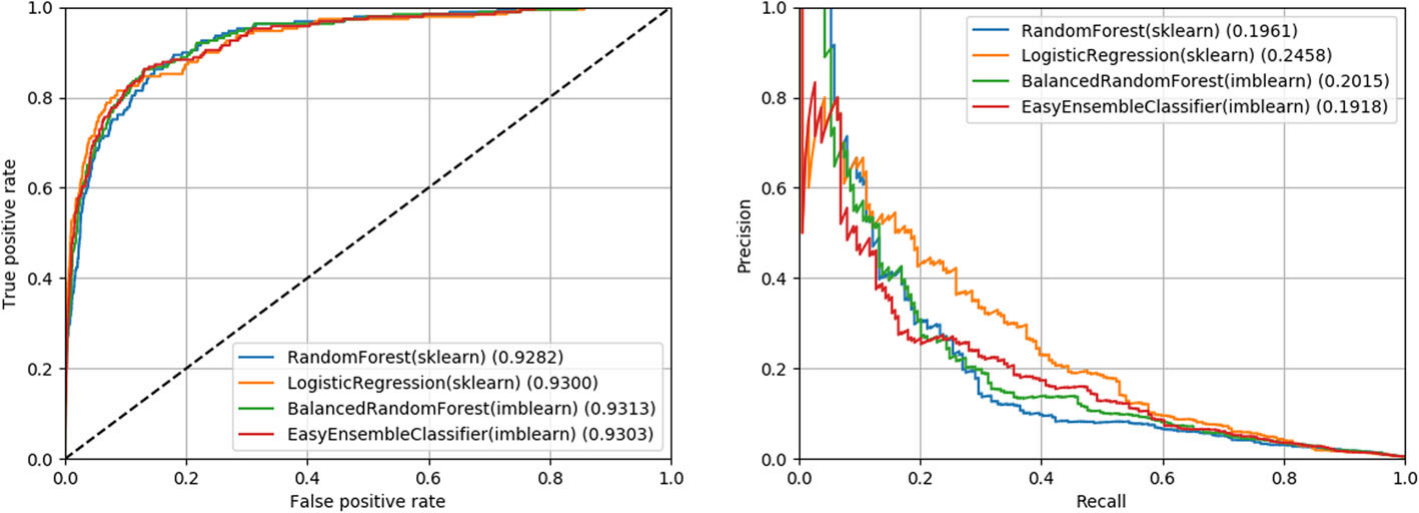
Receiver operator and precision-recall curves. These figures show the performance of the four classifiers on the testing set after hyperparameter tuning and fitting to the training set. On the left, we show the receiver operator curve (false positive rate against the true positive rate). On the right, we show the precision recall curve. Area under the curve (AUROC or AUPRC) is reported beside each method in the legend

The hyperparameters for each classifier were tuned using 10-fold cross validation and the resulting average and standard deviation of balanced accuracy is reported in Table 2. After fitting the tuned classifiers to the full training set, we evaluated the classifiers on the testing set by calculating the area under the receiver operator curve (AUROC) and area under the precision-recall curve (AUPRC) (also shown in Table 2). Figure 1 shows the corresponding receiver operator curves and precision-recall curves for the results from the testing set on all four classifiers.

**Table 2:** Classifier performance statistics

Classifier	CV10 Acc.	AUROC	AUPRC
RandomForest(sklearn)	0.84+-0.13	0.9282	0.1961
LogisticRegression(sklearn)	0.84+-0.13	0.9300	0.2458
BalancedRandomForest(imblearn)	0.86+-0.11	0.9313	0.2015
EasyEnsembleClassifier(imblearn)	0.85+-0.08	0.9303	0.1918

For each tuned classifier, we show performance measures commonly used for classifiers (from left to right): 10-fold cross validation balanced accuracy (CV10 Acc.), area under the receiver operator curve (AUROC), and area under the precision-recall curve (AUPRC). The CV10 Acc. was gathered during hyperparameter tuning by calculating the average and standard deviation of the 10-fold cross validation. AUROC and AUPRC was evaluated on the testing set after hyperparameter tuning and fitting to the full training set

From these metrics, we can see that all four classifiers have a similar performance with regards to AUROC. However, all classifiers have a relatively poor performance from a precision-recall perspective (best AUPRC was 0.2458). This indicates that from a classification perspective, these classifiers would identify a high number of false positives relative to the true positives unless a very conservative cutoff score was used. Practically, we would not recommend using these trained classifiers to do automated reporting because it would either report a large number of false positives or miss a large number of true positives.

### Ranking statistics

We also quantified the performance of each classifier as a ranking system. For each proband, we used the classifiers to calculate the probability of each class (reported or not reported) for each variant and ranked those variants from highest to lowest probability of being reported. We then calculated median and mean rank statistics for the reported variants. Additionally, we quantified the percentage of reported variants that were ranked in the top 1, 10, and 20 variants in each case. While the classifiers were trained as a binary classification system, we stratified the results further to demonstrate differences between variants that were clinically reported as a variant of uncertain significance (VUS), likely pathogenic, and pathogenic.

For comparison, we selected to run Exomiser [33], Phen-Gen [48], and DeepPVP [34]. For each tool, we input the exact same set of phenotype terms used by the classifiers we tested. Additionally, we used the same set of pre-filtered variants from Codicem as input to each ranking algorithm. As a result, all external tools and our trained classifiers are ranking on identical phenotype and variant information.

For Exomiser, we followed the installation on their website to install Exomiser CLI v.11.0.0 along with version 1811 for hg19 data sources. We ran Exomiser twice, once using the default hiPhive prioritizer (incorporates knowledge from human, mouse, and fish) and once using the human only version of the hiPhive prioritizer (this was recommended instead of the PhenIX algorithm [32]). Phen-Gen V1 was run using the pre-compiled binary using the “dominant” and “genomic” modes to maximize the output. Of note, Phen-Gen was the only external method that did not fully rank all variants, so we conservatively assumed that any absent variants were at the next best possible rank. Thus, the reported Phen-Gen comparisons are an optimistic representation for this test data. Finally, DeepPVP v2.1 was run using the instructions available on their website. Details on the exact installation and execution for each external tool can be found in the Additional file 1.

Finally, we added two control scores for comparison: CADD scaled and HPO-cosine. These scores were inputs to each classifier, but also represent two common ways one might naively order variants after filtering (by predicted deleteriousness and by similarity to phenotype). The results for the two control scores, all four external tools, and all four trained classifiers are shown in Tables 3 and 4. A figure visualizing all ranking results can be found in the Additional file 1.

**Table 3:** Ranking performance statistics

Ranking System	Case Rank - Median (Mean)			
	All (n=189)	VUS (n=111)	LP (n=42)	Path. (n=36)
CADD Scaled	57.0 (99.13)	69.0 (107.78)	39.5 (91.24)	28.0 (81.67)
HPO-cosine	22.0 (53.96)	22.0 (56.05)	26.0 (56.38)	19.5 (44.69)
Exomiser(hiPhive)	79.0 (105.34)	85.0 (116.33)	93.5 (101.10)	34.0 (76.42)
Exomiser(hiPhive, human only)	35.0 (53.60)	37.0 (63.84)	34.0 (45.60)	24.5 (31.36)
Phen-Gen	55.0 (48.66)	65.0 (52.91)	47.0 (47.48)	24.0 (36.92)
DeepPVP	15.0 (76.95)	23.0 (79.68)	19.5 (84.95)	6.0 (59.19)
RandomForest(sklearn)	10.0 (29.64)	15.0 (39.27)	8.0 (20.07)	4.0 (11.11)
LogisticRegression(sklearn)	6.0 (29.24)	14.0 (39.87)	3.0 (22.05)	1.0 (4.83)
BalancedRandomForest(imblearn)	8.0 (28.24)	14.0 (38.64)	5.0 (17.67)	3.0 (8.50)
EasyEnsembleClassifier(imblearn)	7.0 (28.72)	15.0 (40.15)	6.0 (18.40)	2.0 (5.50)

This table shows the ranking performance statistics for all methods evaluated on our test set. CADD Scaled and HPO-cosine are single value measures that were used as inputs to the classifiers we tested. The middle four rows (two Exomiser runs, Phen-Gen, and DeepPVP) represent external tools that ranked the same set of variants as the classifier algorithms. Phen-Gen was the only external tool that did not rank every variant in the set, so we conservatively assumed unranked variants were at the next best position despite being unranked. The bottom four rows are the tuned, binary classification methods tested in this paper. Each method was used to rank (prioritize) the Codicem-filtered variants from each proband in the test set, and the position of reported variants was recorded such that lower values indicate better performance with “1” indicating the first variant in the list. The “Case Rank” columns show the median and mean ranks for all reported variants along with the variants split into their reported pathogenicity (variant of uncertain significance (VUS), likely pathogenic (LP), or pathogenic (Path.)) derived from the ACMG guidelines. All values in this table were generated using only the Codicem-filtered variants from testing set

**Table 4:** Top variant statistics. This table shows the ranking performance statistics for all methods evaluated on our test set (same order as Table 3)

Ranking System	Percentage in Top X Variants - X=(1, 10, 20)			
	All (n=189)	VUS (n=111)	LP (n=42)	Path. (n=36)
CADD Scaled	4, 17, 24	0, 9, 15	7, 21, 30	13, 41, 47
HPO-cosine	7, 32, 47	7, 31, 48	7, 28, 40	8, 38, 50
Exomiser(hiPhive)	7, 29, 36	6, 30, 36	2, 16, 28	16, 38, 44
Exomiser(hiPhive, human only)	7, 28, 37	6, 28, 36	2, 16, 30	16, 38, 50
Phen-Gen	4, 21, 30	5, 20, 27	4, 16, 26	2, 27, 44
DeepPVP	11, 42, 52	4, 36, 47	16, 42, 50	27, 61, 72
RandomForest(sklearn)	16, 53, 65	9, 45, 55	19, 61, 76	36, 69, 80
LogisticRegression(sklearn)	23, 58, 72	13, 44, 62	26, 71, 80	52, 88, 94
BalancedRandomForest(imblearn)	16, 55, 67	9, 44, 57	23, 66, 76	33, 77, 86
EasyEnsembleClassifier(imblearn)	17, 58, 70	12, 43, 60	14, 71, 78	36, 88, 94

The “Percentage in Top X Variants” columns show the percentage of reported variants that were found in the top 1, 10, and 20 variants in a case after ranking by the corresponding method

In the overall data, all four classifiers outperform the single-value measures and external tools across the board. Overall, the median rank ranged from 6-10 in the trained classifiers compared to 15 in the best externally tested tool. The classifiers ranked 16-23% of all variants in the first position and 65-72% in the top 20. As one would intuitively expect, all classifiers performed better as the returned pathogenicity increased ranking 33-52% of pathogenic variants in the first position and 80-94% of pathogenic variants in the top 20.

## Discussion

There are two major factors that we believe are influencing the classifiers’ performance relative to the externally tested tools. First, all results were generated using real-world patients from the UDN, but only our four classifiers were trained on real-world patients from the UDN. In contrast, the four external tools were primarily evaluated and/or trained using simulations that do not capture the variation and/or uncertainty that is apparent in the UDN patient datasets. Second, the four classifiers we tested have far more information (i.e. features) available to them than the external tools. As noted in our methods, we tried to reflect an analyst’s view of each variant as much as possible, starting with 95 features that were pruned down to 20 features used by each classifier. Incorporating the same set of features and/or training on real-world patients may improve the externally tested tools with respect to these classifiers.

We expect these classification algorithms could be refined in a variety of ways. First, adding new features could lead to increased performance in the classifiers. Additionally, some of the features represent data that is not freely available to the research community, so replacing those features with publicly accessible sources would likely influence the results. Second, there may be a better classification algorithms for this type of data. The four selected classifiers were all freely available methods intended to handle the large class imbalance in the training set, but other algorithms that aren’t as readily available may have better performance.

Finally, training the classifier on different patient populations will likely yield different results, especially in terms of feature selection and feature importances. The patient phenotypes were gathered from multiple clinical sites, but the reported variants were generated by one clinical laboratory. While there were multiple analysts working each case and a team review process for these cases, we suspect that a classifier trained on results from multiple laboratories would have different results. Furthermore, our classifiers were trained on a wide range of rare disease patients, so restricting to a particular disease type (based on inheritance, phenotype, impacted tissue, etc.) may allow for the classifiers to focus on different feature sets that yield better results.

## Conclusion

We assessed the application of binary classification algorithms for identifying variants that were ultimately returned on a clinical report for rare disease patients. We trained and tested these algorithms using real patient variants and phenotype terms obtained from the Undiagnosed Diseases Network. From a classification perspective, we found that these methods tend to have low precision scores, meaning a high number of false positives were identified by each method. However, when evaluated as a ranking system, all four methods out-performed the single-measure ranking systems and external tools that were tested. The classifiers had median ranks of 6-10 for all reported variants and ranked 65-72% of those variants in the top 20 for the case. For “Pathogenic” variants, the median ranks were 1-4 and 80-94% of those variants were ranked in the top 20 for the case.

Overall, we believe the classifiers trained in VarSight represent a significant step forward in tackling real clinical data. The tested classifiers improved our ability to prioritize variants despite the variability and uncertainty injected by real-world patients. Ultimately, we believe implementing these classifiers will enable analysts to assess the best candidate variants first, allowing for faster clinical throughput and increased automation in the future.

## Additional file

Additional file 1 Supplementary Document. (PDF 673 kb)

## Data Availability

The datasets analyzed during the current study are made available by the UDN in the dbGaP repository under dbGaP accession phs001232.v1.p1. The scripts used to generate all results, figures, tables, and supplements are available on GitHub at https://github.com/HudsonAlpha/VarSight.

## Abbreviations

AUPRC: Area Under the Precision-Recall Curve
AUROC: Area Under the Receiver-Operator Curve
ACMG: American College of Medical genetics
HAIB: HudsonAlpha Institute for Biotechnology
HGMD: Human Gene Mutation Database
HPO: Human Phenotype Ontology
UDN: Undiagnosed Diseases Network
VCF: Variant Call Format
VUS: Variant of Uncertain Significance
